# Long-Term Response and Possible Cure of Patients With B-Cell Malignancies With Dose-Escalated Rituximab

**DOI:** 10.1177/2324709617691307

**Published:** 2017-02-01

**Authors:** Lauren M. Jacobs, Peter H. Wiernik, Janice P. Dutcher, Pablo Muxi

**Affiliations:** 1 George Washington University, Washington, DC, USA; 2 Cancer Research Foundation of New York, Chappaqua, NY, USA; 3 British Hospital, Montevideo, Uruguay

**Keywords:** rituximab, lymphoma, B-cell malignancies, dose escalation, myeloma, Waldenström macroglobulinemia

## Abstract

Rituximab (R), a chimeric monoclonal antibody targeting CD20 antigen on B-cells, has become a standard of care in the treatment of B-cell malignancies, most often in conjunction with cytotoxic chemotherapy. Activity has been demonstrated in many subtypes of B-cell lymphoma, including diffuse large cell lymphoma, follicular lymphoma (FL), mantle cell lymphoma (MCL), chronic lymphocytic leukemia (CLL), lymphocyte-predominant Hodgkin lymphoma, and Waldenström macroglobulinemia (WM). Additionally, dose escalation of R as a single agent has demonstrated improved activity in previously treated/poor prognosis CLL. We present 4 cases of B-cell malignancy (2 CLL variants/MCL, 1 FL, 1 WM) who received dose-escalated R as a single agent and achieved complete response (3 patients) and stable disease/partial response (1 patient) of 6.5+ to 15+ years duration. They have been off treatment for 6.5+ to 15+ years. Toxicity was minimal, with initial infusion reactions similar to those observed with standard dose infusions. There were no serious treatment-related adverse events or infections. Dose escalated R as a single agent may possibly be curative for some patients with B-cell malignancies, unlike the standard empiric dose of 375 mg/m^2^, and deserves further study.

## Introduction

Rituximab (R), a chimeric monoclonal antibody targeting CD20 antigen on B-cells, has become a standard of care in the treatment of B-cell malignancies, most often in conjunction with cytotoxic chemotherapy as induction therapy, and also as a single agent, for induction and/or maintenance therapy.^1-15^ Major activity has been demonstrated in many subtypes of B-cell lymphoma, including diffuse large cell lymphoma,^1,2^ follicular lymphoma (FL),^3-5^ mantle cell lymphoma (MCL),^5-7^ chronic lymphocytic leukemia (CLL),^8-11^ lymphocyte predominant Hodgkin lymphoma (LPHL),^12^ and Waldenström macroglobulinemia (WM),^13-15^ Additionally, dose escalation of R as a single agent has demonstrated improved activity in previously treated/poor prognosis CLL.^10,11^

The rationale for single agent R has been frequently related to providing an effective anti–B-cell lymphoma therapy that causes less hematologic toxicity and thus less risk of infection. It has been studied in elderly patients and patients with comorbidities, and as a salvage treatment after previous combination chemotherapy.^16,17^ A recent report in patients with FL demonstrates that single agent R is of major benefit in that disease, in which patients were treated with a standard 4-week induction course, followed by short-term (4 doses at 2-month intervals) or long-term (maximum 5 years) courses of maintenance.^4^ There was no added benefit from the long-term maintenance compared with the shorter course.

The initial rationale for dose-escalation of R, particularly in patients with CLL, is based on the finding that B-cells from patients with CLL express significantly less CD20 compared with B-cells from patients with FL.^18^ The clinical result of R therapy in patients with small cell lymphoma (tissue equivalent of CLL) in an indolent lymphoma trial using the standard 375 mg/m^2^ induction dose was also inferior to that of patients with FL.^3^ An additional concern that might produce a low response rate in CLL is the large number of circulating cells, with low CD20 expression, and thus diluting out the effectiveness of the antibody.^10^ O’Brien et al conducted a phase I to II clinical trial of dose-escalation R in patients with CLL (n = 40) or other mature B-cell lymphoid malignancies (n = 10, MCL [4], marginal zone [4], prolymphocytic leukemia [(2]).^10^ The first dose for all patients was 375 mg/m^2^ administered over 6 to 12 hours, and for doses 2 to 4, patients received a fixed, but higher dose of R to a maximum dose of 2250 mg/m^2^. The expected first dose toxicity was observed in almost all patients. Other toxicities were observed but were not dose-related. Similarly, Wiernik and Adiga evaluated single agent R in treatment refractory or poor prognosis patients with CLL or MCL variant of CLL (n = 23), administering 4 weekly doses of induction therapy at 375 mg/m^2^/dose, followed by escalation courses with dose escalation within each patient up to 3 g/m^2^/dose.^11^ Some patients then received doses at 2- to 3-month intervals. The overall response rate was 91%, including 64% complete response (CR) and 27% partial response (PR), and the median progression-free survival was 28.5 months.^11^ Of the 23 patients, 9 were treatment naïve. Two of these early patients (patients 1 and 2 here, 20 and 23 of that report) are included in this report of 4 patients with prolonged disease-free survival, including 3 with possible cure following R alone (Table 1). All patients in these reports provided written consent for treatment and publication of their information.

**Table 1.** Summary of Patients.

Patient Age/Gender—Dx/Rx With R	Diagnosis	Confirming Lab	Rituximab Dosing	Response/Duration
41/male—Rx age 42	CLL/MCL variant	t(2;7); CD5+, CD23−, CD20++	375-1500 mg/m^2^	CR × 10+ years
	PB/marrow only		Intermittent, 3 years	
	Binet A			
52/female—Rx age 52	MCL variant	t(11;14); CD5+, CD23−, CD20++	500-1500 mg/m^2^	CR × 15+ years
	PB/marrow only		6 total doses	
	Binet A			
60/female—Rx age 60	FL; bulky left inguinal/iliac LNs	Kappa clonal, BCL6, BCL2− 65% of B cells; Ki67 20%	375-1500 mg/m^2^	CR × 6.5+ years
	Stage IIA		Induction/maintenance × 8 months	
58/female (prior Rx)—Rx with R age 64	WM; marrow/bone/severe anemia; KPS 50%	IgM >400; IgG 173; IgA <7	375-1500 mg/m^2^	PR/SD—no anemia, no clinical findings; Quant Igs stable × 13+ years
	Intermediate risk		1 year	

Abbreviations: Dx, diagnosis; Rx, treatment; CLL, chronic lymphocytic leukemia; MCL, mantle cell lymphoma; CR, complete response; PB, peripheral blood; FL, follicular lymphoma; LN, lymph node; WM, Waldenström macroglobulinemia; KPS, Karnosfky performance score; PR, partial response; SD, stable disease.

## Case Summaries

### Patient 1 (Table 2)

**Table 2.** Course of Patient 1.

Dates	Immunophenotype	Rituximab Treatment
February to October 2001	Lambda/CD5+, CD23−	Dose escalation 375-750 mg/m^2^; monthly maintenance × 1 year, 500-750 mg/m^2^
October 2001	t(2;7)(p11.2,q22)	
January 2003	Kappa/CD5+, CD23+	750 mg/m^2^ every 3 months
September 2003	Lambda/CD5+, CD23−	750 mg/m^2^ every 3 months until April 2004
April 2004 to July 2005	Lambda/CD5+, CD23−	No treatment
July 2005 to March 2006		Re-induction weekly, 750 mg/m^2^ × 4; dose escalation 1000-1500 mg/m^2^, monthly until March 2006
2006-2016	No clonal B lymphocytes	

A 41-year-old previously healthy male presented in February 2001 with an upper respiratory infection and was found to have a lymphocytosis (white blood cell count 12 500/µL, 35% lymphocytes) with an absolute lymphocyte count of 4509/µL. The lymphocytosis persisted despite recovery from his infection. He therefore underwent a hematologic evaluation. Bone marrow biopsy was normocellular with clusters of small interstitial lymphocyte infiltrates. There was trilineage hematopoiesis. This was consistent with a lymphoproliferative disorder. The immunophenotype analysis demonstrated a clonal population of lymphocytes that was lambda light chain+, CD5+, CD23−, bright CD20**+**. The initial assessment did not make a distinction between MCL and variant CLL, although CD23 negativity is consistent with MCL, and this was the working diagnosis. Positron emission tomography (PET) scan in May 2001 demonstrated no lymphadenopathy or abnormal uptake.

Subsequent repeated evaluations of peripheral blood in May and October 2001 continued to demonstrate a clonal population of lymphocytes, all demonstrating a lambda B-cell lymphoproliferative disorder expressing CD5 but not CD23. In October 2001, bone marrow again demonstrated the clonal lymphocyte population with a cytogenetic abnormality of t(2;7)(p11.2;q22), which has been associated with both CLL and marginal zone lymphoma.

He sought treatment in October 2001, after 3 evaluations by different hematologists confirmed the presence of a clonal lymphoproliferative disorder, consistent with MCL or MCL variant of CLL. He requested treatment after investigating MCL prognosis on progression. He still had no evidence of disease outside of peripheral blood and marrow. He was initiated on a treatment strategy of R alone in a dose-escalation approach. He initially received 375 mg/m^2^ weekly × 4 doses, escalated to 500 mg/m^2^ monthly × 3 doses, and then continued every 3 months, initially 500 mg/m^2^ and then 750 mg/m^2^. The dose-escalated R was tolerated well. In January 2003, the immunophenotype analysis of peripheral blood revealed a second clonal B-cell population consisting of cells with kappa light chains that were CD5+ CD23+, consistent with CLL as opposed to the original lambda CD5+ CD23− clone, which was not detected at this evaluation. However, in September 2003 the lambda/CD5+ CD23− clone was again detected but not the kappa clone. He continued R into early 2004. He had a treatment hiatus from April 2004 to July 2005, for personal reasons, and reevaluation of peripheral blood in June 2005 again demonstrated the lambda clone. There was no evidence of disease outside of the marrow/peripheral blood. In July 2005, he restarted R treatment, with further escalation of the dose of R to 1000 mg/m^2^ and 1500 mg/m^2^ as monthly doses through March 2006. In March 2006, the immunophenotype analysis was negative for either clone and has remained so for the subsequent 10 years. He has had annual immunophenotype analyses and physical examinations, and he remains without physical evidence of lymphoma or immunophenotypic evidence of clonal lymphocytosis.

### Patient 2

A 52-year-old previously healthy female was found to have asymptomatic lymphocytosis on a routine clinical evaluation in May 2001. Her white blood cell count was 14 500/µL, 64.7% lymphocytes, with an absolute lymphocyte count of 9360/µL. A computed tomography (CT) scan was normal with no enlarged lymph nodes and no splenomegaly. Immunophenotype analysis of peripheral blood demonstrated a clonal population of B lymphocytes that was CD5+, CD20++, CD23−, confirming a diagnosis of non-Hodgkin’s lymphoma. The initial assessment did not make a distinction between MCL and variant CLL, although CD23 negativity is consistent with MCL, and this was the working diagnosis.

The patient was then referred to our program in July 2001, where the immunophenotype was verified, and she demonstrated a clonal population of lymphocytes in peripheral blood, expressing lambda light chains, CD5+, CD23−, CD20+ bright. The clonal lymphocyte population demonstrated a cytogenetic abnormality of t(11;14)(q13;q32), consistent with MCL or possibly CLL variant.

In July 2001, she was initiated on treatment with R alone, in a dose-escalation approach. The rationale for initiating treatment was concern for possible rapid progression of untreated MCL. She initially received 500 mg/m^2^ (750 mg) for 4 weekly doses, followed by 2 additional doses of 1000 mg and 1500 mg. At that time her immunophenotype was negative for the clonal population of lymphocytes. She did not receive maintenance R. Therapy was tolerated well.

In May 2013, September 2014, and October 2016, immunophenotype analysis of peripheral blood revealed no lymphocytosis, and no clonal population of B-cells. Karyotype was 46,XX without evidence of the t(11;14) translocation. In October 2016, she had normal laboratory evaluations with no lymphocytosis. She remains asymptomatic and without physical evidence of lymphoma nor immunophenotypic nor cytogenetic evidence of clonal lymphocytes in peripheral blood.

### Patient 3

A 60-year-old previously healthy female presented in June 2009 with an edematous left leg, with pain and swelling in the left groin due to palpable lymphadenopathy. She was referred to gynecologic oncology for evaluation of possible gynecologic malignancy. Dilatation and curettage of endometrium did not reveal malignancy. Left inguinal lymph node biopsy revealed FL with kappa-restricted B-cells, CD10 and CD19 positive, comprising 50% to 65% of total cellularity. She developed fever 4 days after biopsy, and CT scan of abdomen and pelvis revealed bulky masses extending from the obturator area into the retroperitoneum, as well as large masses within the pelvis. There were also areas of fluid and gas in the inguinal area and in subcutaneous fat in the left thigh. She was designated stage IIA. As a result of the infection following the nodal biopsy and the continued lymphatic obstruction, the wound required placement of a continuous drain and she received prolonged antibiotics. It was apparent that the wound could not heal until there was significant reduction in lymphadenopathy. She was, therefore, referred for treatment of her newly diagnosed lymphoma.

Because of the ongoing wound drainage and recent infection, she was initially started on therapy with R alone, 375 mg/m^2^ weekly × 4 weeks, with the plan to add cytotoxic chemotherapy after significant wound healing. However, although she demonstrated response, the wound remained open, so R was continued weekly with dose escalation. She continued this with excellent response and eventual wound healing. Chemotherapy was never administered due to resolution of lymphadenopathy with R alone. From January through June 2010, she received monthly maintenance R × 6 doses, escalating to 1500 mg/m^2^. She tolerated this well, with only a first dose infusion reaction. In September 2010, a CT scan revealed no evidence of disease. She continues to have annual scans and physical examinations and laboratory evaluation every 6 months. She remains in complete remission, now 6.5+ years from the end of R therapy.

### Patient 4 (Table 3)

**Table 3.** Course of Patient 4.

Date	Hgb/Hct	Plts × 10^3^	WBC/ANC × 10^3^	β-2 mcG/(Range)	IgM (mg/dL)	IgG (mg/dL)	IgA (mg/dL)
September 2010	14.8/45.5	138	4.2/2.9	2411 (0-2301)	412	173 (lln 844)	<7
May 2012	15.3/47.5	161	3.9	2400 (0-2300)			
April 18, 2013^a^					398 (range 50-196)	123 (range 844-1912)	<7 (range 68-423)
February 9, 2015, to March 2, 2015^b^	15.3/45.2	145	4.5/2.5	2335 (0-2300)	371 (range 50-196)	112 (range 844-1912)	<28 (range 68-423)
July 1, 2016	15.7/47.5	150	4.7/2.8	2246 (0-2300)	365 (range 50-196)	116 (range 844-1912)	<6.1 (range 68-423)

Abbreviations: Hgb, hemoglobin; Hct, hematocrit; WBC, white blood cell; ANC, absolute neutrophil count.

a April 18, 2013: SPEP: total protein 5.8 g/dL (range 5.8-8.3); M-spike 1.9% IgM Kappa.

b March 2015: SPEP TP 5.9, albumin 4.4; M-spike 1.4% IgM Kappa.

In 1997, a 58-year-old woman was diagnosed with symptomatic WM for which she was treated with corticosteroids and an alkylating agent, and she achieved clinical remission with no further treatment. In 2003 (age 64), she became severely anemic and very weak, with Karnofsky performance status of 50%, and she was found to have a bone lesion thought to be consistent with her disease. Quantitative immunoglobulin studies demonstrated elevated IgM, and subnormal IgG and IgA. She did not have symptoms of hyperviscosity, but she required multiple red cell transfusions. She was treated for 1 year with escalating doses of R alone, starting at 375 mg/m^2^ weekly × 4 weeks as induction therapy, followed by escalation of monthly maintenance doses to a maximum of 1500 mg/m^2^. She tolerated treatment well and did not have an IgM flare with therapy. Her last dose was 1500 mg/m^2^ in December 2003, by which time she had become clinically well and fully functional and transfusion independent. No treatment was administered from that point, and she was completely well until she developed a submandibular mass in November 2009 (age 70). A biopsy confirmed lymphoplasmacytic lymphoma (WM). Her bone marrow demonstrated hypocellularity with trilineage hematopoiesis and a 10% monoclonal atypical lymphoid population expressing cytoplasmic IgM Kappa.

PET/CT scan in November 2009 showed only the submandibular mass with extension to the right soft palate. This localized recurrence was treated with external beam radiation with complete resolution of the mass. She received no additional systemic treatment. A follow-up PET/CT scan in late 2010 showed no new masses and she remained stable off therapy. She received iron-chelating agents periodically following her multiple transfusions in 2003.

She has had no systemic treatment since 2003, and her laboratory values have remained stable (see below). She remains well, and fully active, without infections or major medical issues. She has laboratory manifestations of WM (elevated IgM and suppressed IgG and IgA); however, her blood counts of hemoglobin, platelets, white blood cells, and chemistry of total protein and albumin remain within normal range. Her quantitative immunoglobulins have been stable for more than 10 years.

## Discussion

The use of R therapy at the standard dose (375 mg/m^2^) has become a mainstay of management of B-cell malignancies, such that it is combined with all first-line chemotherapy regimens and is used as a single agent to reduce toxicity in vulnerable populations and as maintenance in many clinical situations.^1-15^ As noted, a recent report demonstrates excellent outcome in patients with FL treated with single agent induction and followed by a short course of maintenance.^4^ Single agent and maintenance R is also widely used in patients with WM.^13-15^ Additionally, the combinations of R plus combination chemotherapy with cyclophosphamide, doxorubicin, vincristine, prednisone (CHOP) or bendamustine have produced major benefit in patients with MCL.^5,7^ However, it is important to note that the standard dose of 375 mg/m^2^ of R was developed empirically, to allow enrollment and completion of a clinical trial of sufficient power to demonstrate the benefit of this agent, which led to approval of the drug.

Therefore, it is also important to review the data from 2 early reports of dose escalation of R in patients with CLL, in which patients initiated therapy at the first dose of 375 mg/m^2^, followed by increased doses in subsequent cycles. In the O’Brien report, patients remained on the same dose after the first escalation.^10^ Patients with white blood cell counts greater than 100 × 10^9^ had significant toxicity with initial doses. One of 4 patients with MCL (the other 3 did not complete therapy), 2 of 2 patients with prolymphocytic leukemia, and 3 of 4 patients with marginal zone leukemia had partial responses.^10^ Thirty-nine patients with CLL were assessable for response, and there were 14 PRs. Among the CLL patients, there was a dose-response relationship, in that 9/14 PRs were achieved at doses of 1000 mg/m^2^ or greater.^10^ In the report by Wiernik and Adiga, with intrapatient dose escalation, among 23 patients with CLL (including 2 with MCL features), the overall response rate was 91%, including 64% CR and 27% PR as of the report in 2011.^11^ There were 9 treatment-naïve patients among that group, and at the time of the report, their median duration of response (DR) was 24+ months, and their median progression-free survival (PFS) was 25+ months.^11^ Among the 14 fludarabine-naïve patients, the median DR was 29+ months and median PFS was 31+ months at the time of that report.^11^ Additional follow-up of the entire group is not available.

We are now reporting long-term responses among 4 patients who received dose-escalated R alone, 2 for poor prognosis CLL (MCL variants by immunophenotype [2], and by cytogenetics [1], variant cytogenetics [1], and with a second clone [1]), one for FL in a clinical situation in which chemotherapy was initially contraindicated, and one for poor prognosis, refractory WM (Table 1). Three patients were treatment naïve, and one had been previously treated with chemotherapy. Of note is that the patient with MCL variant by immunophenotype and t(11,14) cytogenetics also strongly expressed CD20, and obtained a CR after only 6 doses of R, with doses of 750 mg to 1500 mg. She has remained with CR for 15+ years. The second MCL/CLL patient (MCL immunophenotype, but variant cytogenetics, and a second CLL-like clone) received initially lower doses (375 mg/m^2^ × 4 doses, then every 3 months maintenance at 500-750 mg/m^2^; Table 2). Two years later, the MCL immunophenotype clone reappeared. He remained with only PB involvement, and was retreated with higher doses of rituximab 4 years after diagnosis, with doses of 1000 to 1500 mg/m^2^, monthly for 1 year, and achieved a CR that has continued for 10+ years. The FL patient received 8 months of treatment with R with doses of 375 mg/m^2^ escalated to 1500 mg/m^2^, achieving a CR, as her open wound healed. In view of the CR, no chemotherapy was added. She has remained in CR for 6.5+ years. The patient with WM has had a prolonged disease course, treated initially with chemotherapy, 6 years before recurrence with clinically significant and refractory disease. After failing additional chemotherapy, she was treated with single agent dose-escalated R for 1 year, and achieved PR/SD with no clinical features of illness, and resolution of anemia and stable, abnormal serum immunoglobulins and normal beta-2 microglobulin. She has remained with clinical PR for 13+ years.

In summary, with renewed interest and modern reports of major benefits from single agent and short maintenance R in patients with B-cell malignancies, the option of dose escalation must be reexamined as part of the clinical armamentarium. The dose-escalation approach has produced long-term response in these 4 patients and may have been curative in 3 of them. Additionally, the only toxicity experienced was infusion reaction with the initial dose, and no long-term toxicities have been observed to date. This approach deserves further study.
